# Down-regulation of tissue factor inhibits invasion and metastasis of non-small cell lung cancer

**DOI:** 10.7150/jca.37321

**Published:** 2020-01-01

**Authors:** Qing Xia, Xu Zhang, Qianqian Chen, Xiangyun Chen, Junliang Teng, Changhui Wang, Ming Li, Lihong Fan

**Affiliations:** 1 Department of Respiratory Medicine, Shanghai 10th People's Hospital, Tongji University, Shanghai 200072, China.; 2 Institute of Energy Metabolism and Health, Tongji University School of Medicine, Shanghai, China.; 3 Institute of Development and Research of Holistic Integrative Medicine, Tongji University, Shanghai, China.; 4 School of information management and engineering, Shanghai University of Finance and Economics, Shanghai, China.

**Keywords:** non-small cell lung cancer (NSCLC), tissue factor, prognosis, biomarker

## Abstract

**Objective:** Tissue factor (TF) is clinically identified as a marker for the detection of various types of cancer as well as the prediction of prognosis for cancer patients. This present study aims to explore the possibility and feasibility to use plasma TF as a biomarker for the prediction of prognosis of patients with non-small cell lung cancer (NSCLC).

**Methods:** A total of 100 patients with NSCLC at stage I to IV was included in the study, in whom the expression of plasma TF was detected. The Cox proportional-hazards regression model was then used to analyze the collected information, attempting to identify how patients' overall survival (OS) was associated with the expression of plasma TF. To verify the function of TF in invasion and metastasis, the expression of plasma TF was downregulated by SiRNA both *in vivo* and *in vitro*.

**Results:** The expression of plasma TF in NSCLC patients was related to the diagnosis age of the patient. It was noted that patients with high TF expression levels tended to have worse OS performance, which implied that TF could be used as a marker for patients with stage I-IV NSCLC (HR = 2.030, 95% CI = 1.21-3.398, *P* = 0.007). TF down-regulation inhibited the growth of tumor *in vitro* as well as the metastasis and invasion of NSCLC cells *in vivo*.

**Conclusion:** Both *in vivo* and *in vitro*, the invasion and migration of NSCLC cells are suppressed by TF knockdown. TF has the potential to become an effective biomarker for the prediction of prognosis of patients with stage I-IV NSCLC.

## Introduction

As a seriously fatal malignancy, lung cancer has become a common type of cancer all over the world, among which 80% of lung cancer patients are diagnosed with non-small cell lung cancer (NSCLC). Approximately 67% of patients with NSCLC have lost their opportunity for radical surgery because of delayed diagnosis, and an overwhelming number of NSCLC patients with early diagnosis who have undergone surgery are also at risk of recurrence or distant metastasis 1. At present, there is no reliable marker to predict the prognosis of NSCLC patients.

A hypercoagulable state is currently believed to be closely associated with tumor progression 2, and some important blood coagulation factors have been demonstrated to have a good clinical value in early diagnosis, therapeutic planning and prognostic prediction of cancers.

Tissue factor (TF) refers to an important factor that initiates the extrinsic coagulation cascade that can activate downstream signaling by binding to factor VIIa (FVIIa), thus leading to platelet activation, fibrin deposition and thromblin generation 3. But most previous studies about TF mainly focused on the physiology processes, knowing that TF plays an important role in the development embryo and normal hemostasis 4, 5. Although there are some studies reporting the relationship between TF and inflammation 6, how TF and cancer interact remains obscure.

It was recently reported that inhibition of TF could blunt osteosarcoma progression 7, and patients with ovarian cancer, who had high expression of TF, demonstrated poor prognosis as compared with those with a low TF expression 8. Similar findings were also reported in breast cancer, prostate cancer and pancreatic cancer 9-12. However, the relationship between TF and NSCLC remains elusive.

This present study aims to investigate how TF could affect the prognosis of NSCLC *in vitro* and *in vivo*. The findings implied a significant correlation between the expression of TF and the T status and TNM stage in clinical NSCLC samples, suggesting that it is of great potential to be used as an effective biomarker for prognostic prediction of NSCLC patients. In addition, the downregulated TF by siRNA had an inhibitory effect on the growth of tumor *in vitro* and the migration and invasion of NSCLC cells *in vivo*.

## Materials and Methods

### Clinical samples and cell lines

Included in this study were 100 NSCLC patients that had been treated in the Department of Respiratory Diseases of Shanghai Tenth People's Hospital (Shanghai, China). The collection of plasma samples was performed on these patients under informed consents, and these samples were snap-frozen right after collection at -80˚C. It was confirmed that all the sampled patients were exempted from radiotherapy or chemotherapy before they had the surgery performed on them. PC9 AND A549, the experimental cell lines of human NSCLC, were bought from the Chinese Academy of Sciences (Shanghai, China). Dulbecco's modified Eagles's medium (DMEM) (Gibco, Grand Island, NY, USA) with additives of streptomycin (100 μg/ml) (Enpromise, Hangzhou, China), penicillin (100U/ml) and 10% fetal bovine serum (FBS) (Gibco, Shanghai, China) was used to maintain these cells. The experimental cells were all moved into a moist environment with 5% CO_2_ for incubation at a constant temperature of 37˚C.

### ELISA

The exaction of plasma samples was performed after the 10-hour starvation treatment before angiography. These samples were subject to a 10-minute centrifugation at 1000g, and then maintained in a low-temperature (-80°C) environment for later use. An ELISA kit (Abcam, Shanghai, China) was used for the measurement of the TF levels of the plasma.

### Transfection assay

The chemosynthesis of TF siRNA as well as its negative control (NC) was performed in Shanghai Genepharma Co., Ltd. (Shanghai, China). The experimental cells (1x106) were cultured in a 6-well plate, with each well added with DMEM (which was antibiotic- and serum-free). After lung cancer cells reached 30-50% confluency, Lipofectamine 3000 (Invitrogen, Carlsbad, CA, USA) was used to infect these cells with with TF-siRNA or NC at working concentrations strictly following the instructions of the manufacturer. After incubating these cells for 4 to 5 hours, the original DMEM was removed and new DMEM supplemented with 10% FBS was added. Afterwards, these cells were all subject to a 24-hour incubation in an environment with a certain concentration of CO_2_ at a constant temperature of 37˚C before any further test.

### RNA isolation and qRT-PCR from tissues

Trizol reagent (Invitrogen, Carlsbad, CA) was used to extract complete RNAs from PC9 and A549 cell lines strictly following the instructions of the manufacturer. Concisely, 10-volume TRI Reagent was applied to the homogenization of the samples, which was then subject to a 10-minute centrifugation at 12,000xg at 4°C. A clean tube was used to hold the supernatant, and100 μL BCP per 1 mL of TRI Reagent solution was added into the tube to homogenize the supernatant. The ethanol was removed from the supernatant via a 5-minute centrifugation at 7,500g. Then, the RNA pellet was put in open air to dry spontaneously. THE RNA Storage Solution was used to dissolve the extracted complete RNAs. The PrimeScript RT Master Mix (Perfect Real Time) kit was applied to the synthesis of cDNA for RT-PCR. β-actin was used as an internal control. TF primers used were: Forward 5'-CAAACCCGTCAATCAAGT-3' and Reverse 5'-CTTCACATCCTTCACAATCTCG-3'. β-actin primers used were: Forward 5'-ATCATGTTTGAGACCTTCA ACA-3' and Reverse 5'-CATCTCTTGCTCGAAGTCC A-3'. qRT-PCR was performed in an ABI Prism 7600 system (Applied Biosystems, USA) strictly following the instructions of the manufacturer, subject to 10-minute cycling at 95˚C and the subsequent 45 cycles for 15s and 45s at 95˚C and 65˚C, respectively. The 2-ΔΔCt method was applied to the calculation of the data about gene expression. All experiments were done in triplicate.

### Cell proliferation assay (CCK-8 assay)

Cells were pipetted onto a 96-well plate in triplicate at 5.0×10^4^ in each plate on the first day (24 hours after the transfection of siRNA). The 10 ll WST-8 from Cell counting kit-8 (Dojindo, Shanghai, Japan) was applied to the measurement of the absorbance at 450nm every other day after the samples were incubated for one hour until the 6^th^ day. The control group was cells without siRNA transfection. The author totally conducted three independent experiments.

### Colony formation assay

Four hours after being transfected, the cells (300 cells from each group) were moved to a 6-well plate added with complete medium for further incubation. The equal distribution of the cells was achieved by shaking the plate. About 8 days later, when the colonies could be seen with bare eyes, the culture medium was removed and PBS was used to wash the plate thoroughly. Afterwards, 95% ethanol was used to fix these colonies for 10 minutes, and 0.1% crystal violet was then used to stain these cells for another 10 minutes after they were thoroughly dried. Next, pure water was used to wash the plate for three times, and after the number of cells contained in the well reached up to 50, the author counted the colonies observable. This experiment was performed in triplicate.

### Wound-healing assay

After transfection, the cells were move to a 6-well plate for incubation to get a confluence of around 90%. Then, a sterile pipette tip was used to make a scratch in each of the six wells. A light microwave was used to observe wound-healing after the plate was washed in PBS. Pictures were taken at 0, 12, 24 and 48 h, respectively during the healing process. This operation was performed twice, and pictures with sufficient representativeness have been selected and demonstrated.

### Transwell invasion and metastasis assay

Chemicon Cell Invasion Assay kit (Chemicon, Temecula, CA, USA) was used to perform a Transwell invasion assay. The top chamber with a Matrigel (2 mg/ml)-coated membrane containing 8-mm diameter pores in 200 μl serum-free DMEM contained the TF-siRNA or NC transfected cells (5x104 cells/Transwell), while the lower chambers were added with 500 μl DMEM containing 10% FBS. 0.1% crystal violet was used to stain the membrane after it was incubated for 48 consecutive hours, and the stained membrane was put under a microscope for observation after the removal of cells and Matrigel in the top chamber. The author totally selected five spots in each membrane on a random basis, and the cells in each spot, which had penetrated the membrane, well counted at a magnification of x200. The number of cells that had invaded the membrane was used as an indicator of the cells' invasion ability. All the experiments were performed in triplicate. Similarly, the metastasis assay was also conducted except that on the top chamber, there was no Matrigel-coated membrane.

### Western blotting

Ice-cold PBS was used to wash the cells, RIPA lysis buffer (100 μl/well, Beyotime) was used to re-suspend the cells afterwards. Then, these cells were subject to a 30-minute centrifugation at 4˚C (Eppendorf 5804R, Eppendorf Biotech, Hamburg, Germany). A BCA protein assay kit (Beyotime) was used to quantitate the concentration of the protein in the collected supernatant. 5X SDS loading buffer (Beyotime) was used to denature the protein samples for 10 minutes at the temperature of 100˚C. 8% sodium dodecyl sulfate polyacrylamide gel electrophoresis (SDS-PAGE, Beyotime) was used to separate the extracted compete protein which was then moved to a 0.45-μm nitrocellulose membrane (Beyotime). Primary antibodies against TF (abcom, Shanghai, China), and β-actin (1:1,000 dilution; sc-1616-R; Santa Cruz Biotechnology, Inc., Santa Cruz, CA, USA) were used for the incubation of the membrane at the temperature of 4˚C. Then, the membrane was washed thoroughly in PBST (Shanghai Engineering Co.), and secondary antibodies were used to incubate the membrane for another hour. An Odyssey Scanning system was used to detect the immuneractive protein.

### Statistical Analysis

Data collected from at least three independent experiments are expressed as the means ± standard deviation (SD). Different aspects among all the experiment groups were evaluated via the Student's t-test in SPSS 20.0 software. χ2 test was used for categorical data. The Kaplan-Meier method was applied to the estimation of patients' survival curves, and the log-rank test was run to assess the different distributions of these curves. The prognostic hazard ratio was estimated using the Cox proportional-hazards regression model. Results with a P-value under 0.05 were considered statistically significant.

## Results

### Plasma TF expression is associated with the age at the time of diagnosis of NSCLC patients

On the basis of the subgroup classification, the relationship between NSCLC patients' clinicopathologic features and the expression level of TF was analyzed. **Table 1** has demonstrated all the results. Patients aged 65 years and above were more likely to have higher levels of TF expression (≤ 65* vs.* >65, *P* = 0.006), indicating that there was a significantly strong relationship between TF expression and the age at the time of diagnosis. No statistically significant correlation of TF was observed with gender (*P* = 0.107), the smoking status (*P* = 0.317), the histological type (*P* = 0.603), the T status (P = 137) the N status (*P* = 0.499), stage (P = 0.062), differentiation (*P* = 0.829) or the EGFR mutation status (*P* = 0.840).

### TF expression is correlated with overall survival (OS) of NSCLC patients

**Figure 1:**
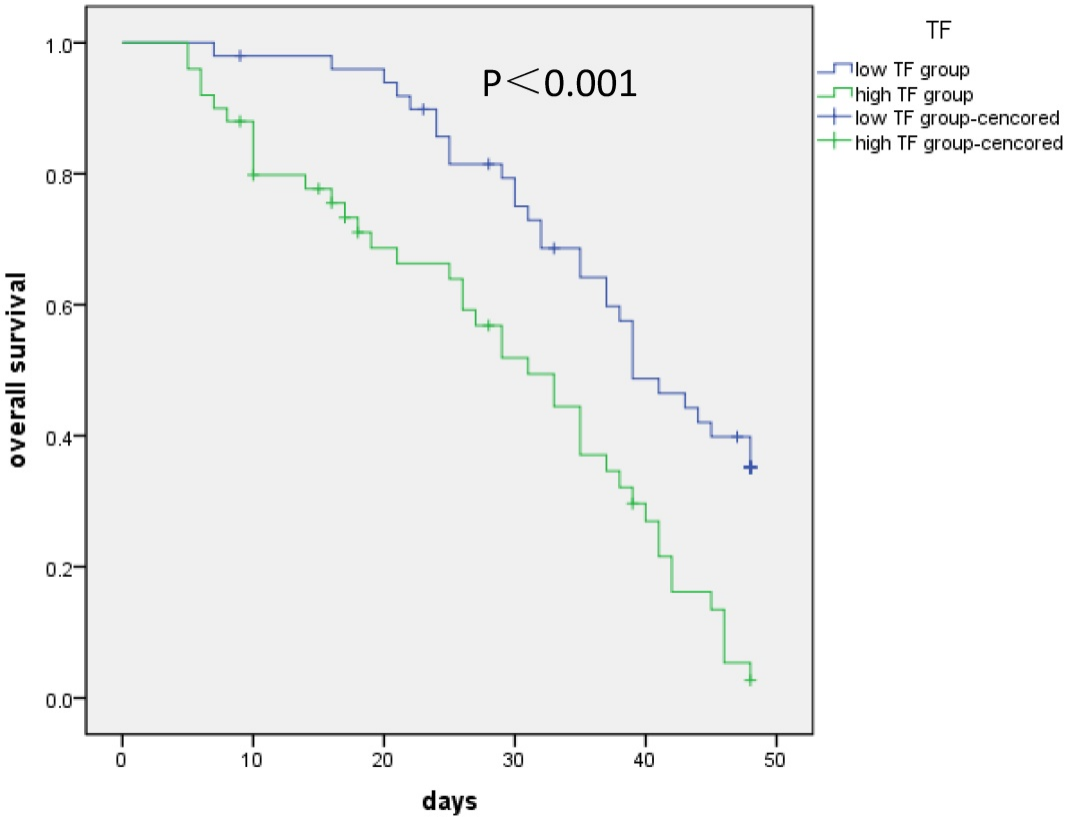
** Correlation of TF expression with overall survival of patients.** Kaplan-Meier analysis was conducted to evaluate how NSCLC patients' OS performance was correlated with the expression levels of the TF in their cells. Higher TF (more than median value) expression of these patients were generally associated with lower OS performance (log-rank test: *P* < 0.001).

Kaplan-Meier analysis was conducted to evaluate how NSCLC patients' OS performance was correlated with the expression levels of the TF in their cells. According to the results, higher TF expression of these patients were generally associated with lower OS performance (log-rank test: *P* < 0.001) (**Figure 1**). The analysis of the collected survival data of these patients was conducted with the cox proportional hazards regression to test the independence of TF expression as a prognostic risk factor. Based on the results of the univariate regression analysis, patients with poor OS performance generally had significantly high levels of TF expression, a high T (T3/4) and TNM stage (*P* < 0.001) (**Table 2**). Based on the results of the multivariate analysis, there is a significantly negative correlation between the OS performance of patients and their TF expression levels (HR = 2.030, 95% CI = 1.212-3.398, *P* = 0.007). Generally, it is safe to say that the expression level of plasma TF in patients with I-IV stage NSCLC can be used as an independent biomarkers for their prognosis.

With these findings, we conducted further validation *in vivo* and vitro experiments.

### TF knockdown decreases the proliferation ability of NSCLC cells *in vitro*

**Figure 2:**
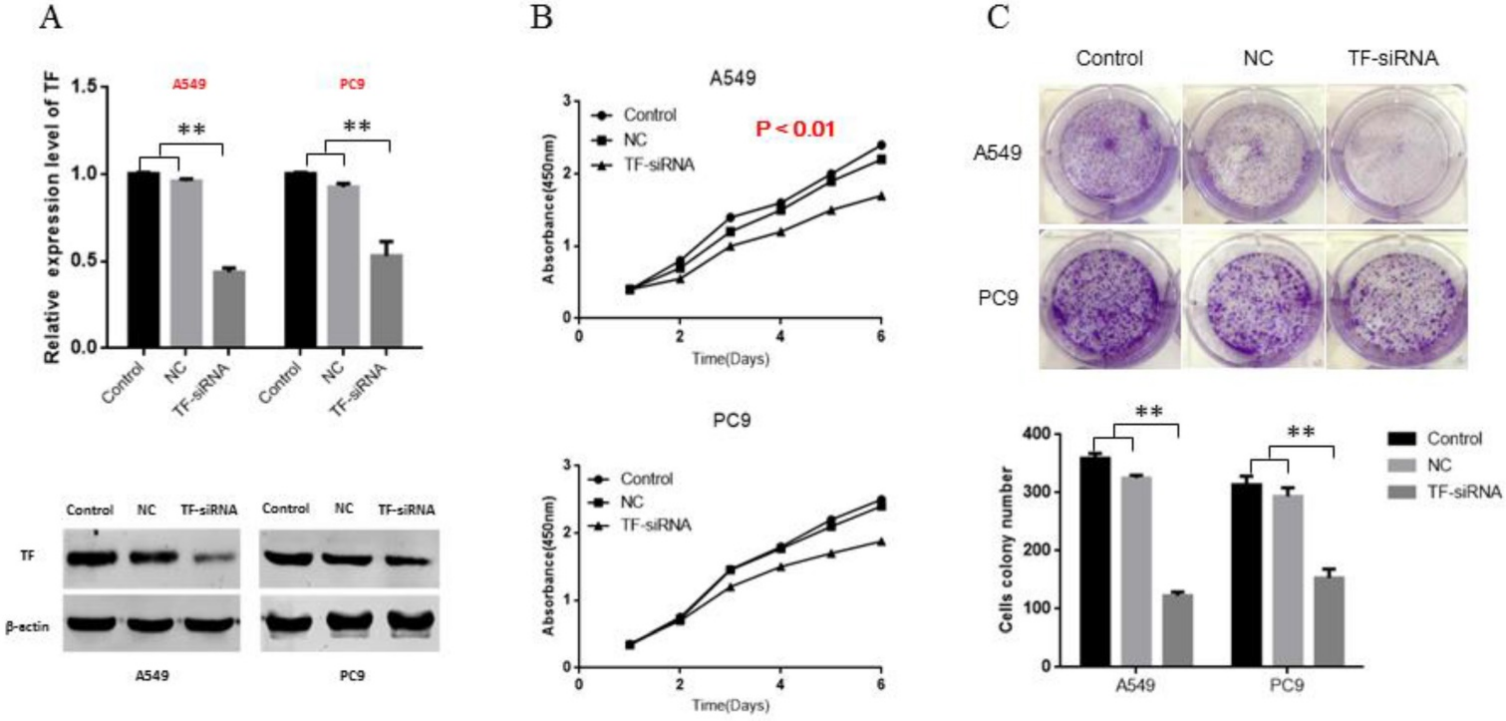
** TF knockdown decreases proliferation ability of NSCLC cells *in vitro*.** (A) siRNA suppressed the TF mRNA and protein expression in NSCLC cells. (B) knockdown of TF inhibit the proliferation ability of NSCLC cells. (C) knockdown of TF inhibit the colony formation ability of NSCLC cells.

To explore the role of TF in NSCLC, siRNA of TF was transfected, and siRNA-NC was used as negative control. Following transfection, knockdown of TF was confirmed by qRT-PCR and Western blot (**Figure 2A**). Based on the cell proliferation assay (CCK-8), of proliferation of both PC9 and A549 cells were significantly inhibited by the knockdown of TF expression (p<0.01) (**Figure 2B**). Based on the results of the colony formation assays, the number of colonies in the NC group was significantly larger than that in the siRNA-transfection group (p<0.01) (**Figure 2C**).

### TF knockdown suppresses NSCLC cell migration and invasion *in vitro*

**Figure 3:**
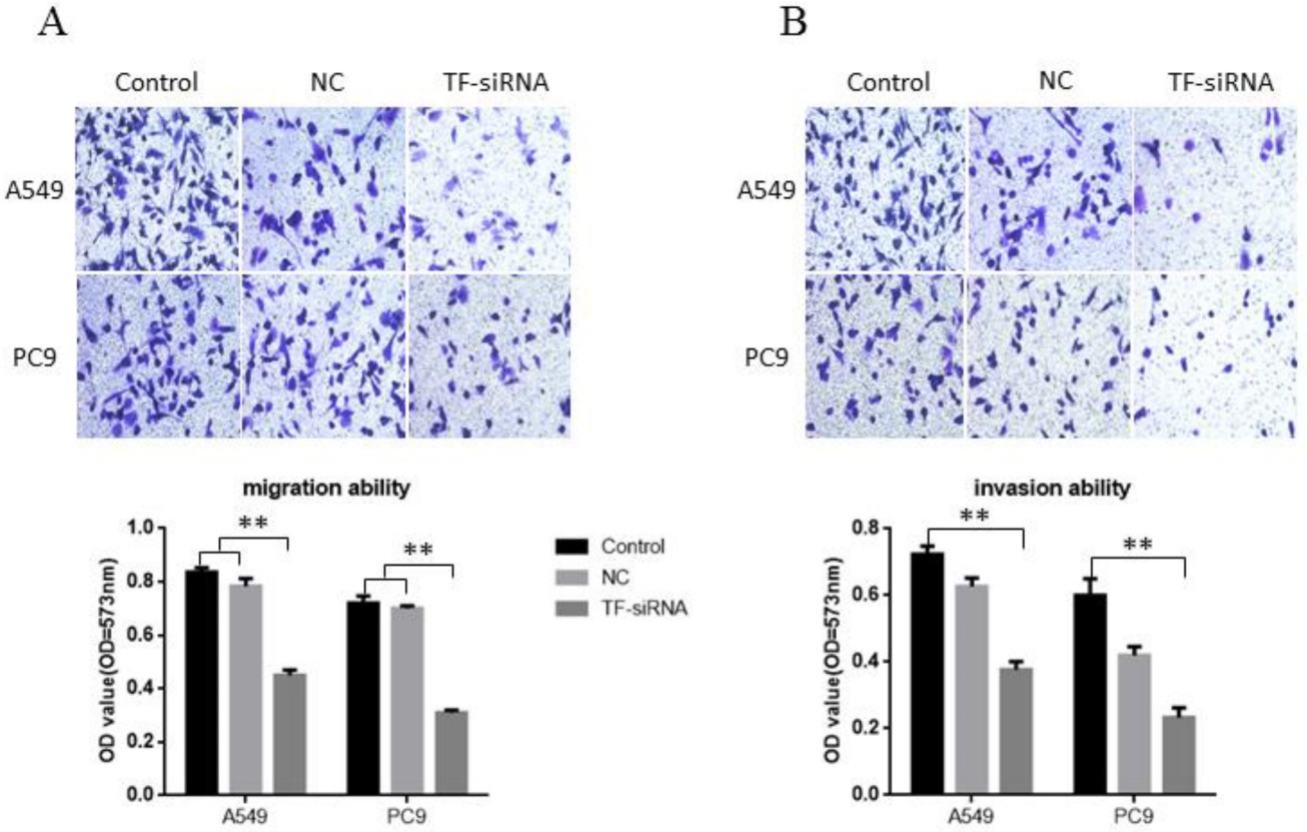
** TF knockdown suppresses NSCLC cell migration and invasion *in vitro*. (A)** knockdown of TF inhibit the **migration ability** of NSCLC cells. (B) knockdown of TF with si-TF inhibit the **invasion ability** of NSCLC cells.

Transwell without Matrigel and Transwell with Matrigel were both conducted to explore the potential of TF expression to affect the migration and invasion of NSCLC cells, respectively. It was found that decreased expression of TF impeded A549 cell migration by 46.21% and PC9 cell migration by 56.94% when it was knocked down by siRNA (**Figure 3A**). Similarly, A549 cell and PC9 cell invasion was also reduced by 47.92% and 61.1% by siRNA respectively (**Figure 3B**). These findings support the conclusion that TF has a significantly strong promoting effect on the invasion and migration of human HSCLC cells.

### TF knockdown suppresses NSCLC tumor growth *in vivo*

**Figure 4:**
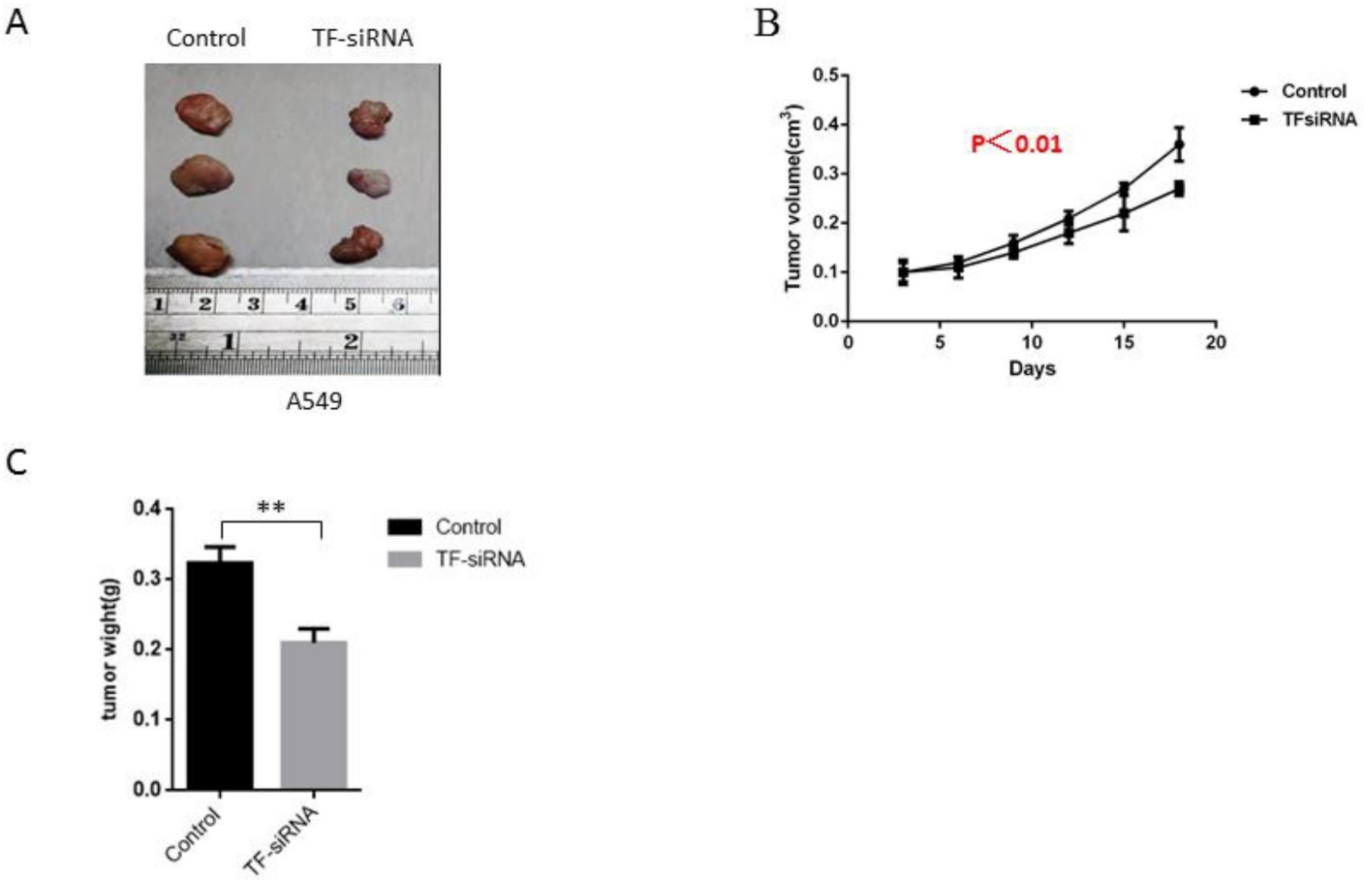
** TF knockdown suppresses NSCLC tumor growth *in vivo*.** (A) experiments were conducted on male nude mice that were inoculated with TF-siRNA-transfected PC9 and A549 cells or empty vectors. TF knockdown inhibited tumor growth 21 days after injection. (B) control group shares more tumor weight than TF-siRNA transfected group. (C) control group shares bigger tumor size than TF-siRNA transfected group.

For the further exploration of the ability of TF expression knockdown to influence the growth of tumor *in vivo*, experiments were conducted on male nude mice that were inoculated with TF-siRNA-transfected A549 cells or empty vectors. It was found that TF knockdown dramatically inhibited tumor growth 21 days after injection, as demonstrated by substantial reduction in body weight and tumor size of the experimental animals (**Figure 4A,B,C**), indicating that TF downregulation was able to reduce NSCLC growth *in vivo*.

## Discussion

The present study explored the potential clinical value of plasma TF expression and discovered that plasma TF could be used for the prognostic prediction for NSCLC patients. A significantly higher level of TF expression was detected in the plasma of stage III-IV NSCLCL patients than stage I-II NSCLC patients. Additionally, a high TF level was generally correlated with poor OS performance, and therefore TF expression may be applied as an effective independent marker for NSCLC patients' prognosis. According to the analysis of the expression patterns of plasma TF as well as the correlation between plasma TF expression and NSCLC patients' prognosis and clinicopathologic features, we further conducted experiments both *in vitro* and *in vivo*, and found TF knockdown could inhibit the ability of NSCLC cell proliferation, invasion and migration.

As a transmembrane receptor of glycoprotein, TF serves as an important factor in cells to initiate the extrinsic pathway of the blood coagulation cascade and exerts its primary function through a natural high affinity interaction with its gland, factor VII (fVII), and its activated form, fVIIa 13. Recent studies about TF were conducted in pancreatic, breast, head and neck squamous cell carcinoma, and colorectal tumor cell lines 8, 14-17. Scholars who study the relationship between cancer and coagulation have been focused on the investigation in TF. It has been reported that patients with cancer are very likely to develop venous thromboembolism (VTE), such as deep vein thrombosis and pulmonary embolism, which is regarded as a notable predictor of decreased 2-year survival 18. The incidence of VTE among cancer patients is six times higher than that among normal population 19. VTE is identified as a great risk of mortality for patients with cancer, and a certain number of potential biomarkers is now identified to be associated with higher risks for thrombosis, especially TF, which has gained increasing attention. The mechanism research about how TF is of great importance in the development of cancer demonstrated that TF could regulate MAPK, PI3K 20 and EGFR pathways, and this process was promoted during epithelial-to-mesenchymal transition (EMT) 21, thus inducing the release of vascular endothelial growth Factor (VEGF) from tumor 22-24. In addition hypoxia could also induce high TF expression 25, 26. Magnus et al (30) reported that TF could induce escape from tumor dormancy and genetic mutations. All these studies favored the conclusion that TF is of great significance in the development of several types of cancers, NSCLC included.

The present study presents some advancement compared with previous literature. First of all, hospital-based patient samples were included in the study, together with long follow-up periods, which increased the validity of the collected data. In addition, patients with PE and DVT were not included in the sample selection to avoid possible impact on TF expression. This study has been the first literature to identify TF as an independent prognostic factor of OS for lung cancer patients, which suggests the huge potential of TF to be an effective biomarker for the prediction of the prognosis and recurrence in terms of OS in NSCLC patients.

Nevertheless, the study is also limited in some aspects. For example, the postoperative chemotherapy regimens present high heterogeneity, and the clinical information is relatively limited. Additionally, it was nearly impossible to avoid the bias in the selection of samples. Larger-sample clinical studies need to be conducted to further verify the findings in the present study.

In conclusion, our study demonstrated the inhibitory effect of TF knockdown on the migration, invasion and proliferation of NSCLC cells *in vitro*, and plasma TF level could be used as an effective non-invasive marker for the prediction of NSCLC patients' prognosis. In our ongoing study, we will build a TF knockout mouse model to further validate the above conclusions.

## Figures and Tables

**Table 1:** Association between TF expression in plasma and patients' characteristics.

Factors	Plasma TF(n=100)		p
	Low(n=50)	High(n=50)	
**Age(years**)			0.006
≤65	31	43	
>65	19	7	
**Gender**			0.107
Male	24	32	
Female	26	18	
**Smoking status**			0.317
Nonsmoker	28	23	
Ever-smoker	22	27	
**Histologic type**			0.603
Non-squamous	42	40	
Squamous	8	10	
**T status**			0.137
T1-2	46	41	
T3-4	4	9	
**N status**			0.499
N0	38	35	
N1-3	12	15	
**Stage**			0.062
I-II	45	38	
III-IV	5	12	
**Differentiation**			0.829
Well,moderate	34	35	
poor	16	15	
**EGFR mutation status**			0.840
Mutated	21	22	
Wild type	29	28	

**Table 2:** Univariate and multivariate analyses for overall survival in patients with NSCLC.

Variables	univariate	multivariate	Variables	univariate	multivariate	Variables
	HR	95%CI		HR	95%CI	
Age,years (>65 vs ≤65)	1.396	0.825-2.360	0.213	-	-	-
Gender (male vs female)	1.648	1.019-2.665	0.042	0.918	0.518-1.627	0.769
Smoking (YES vs NO)	0.946	0.594-1.507	0.816	-	-	-
Histology(squamous vs non-squamous)	2.015	1.148-3.56	0.015	1.189	0.565-2.502	0.648
Pathological T (T3-4 vs T1-2)	4.488	2.377-8.473	<0.001	3.211	1.440-7.160	0.004
Lymph node metastasis (N1-3vs N0)	1.865	1.125-3.092	0.016	1.388	0.756-2.550	0.290
Differentiation (poor VS well, moderate)	1.107	0.669-1.833	0.693	-	-	-
EGFR mutation status (NO vs YES)	1.337	0.830-2.154	0.233	-	-	-
Staging (III, IV vs I, II)	7.104	3.705-13.622	<0.001	5.168	2.353-11.352	-
TF in plasma (high vs low)	2.512	1.551-4.068	<0.001	2.030	1.212-3.398	0.007

## Abbreviations

TF: tissue factor
NSCLC: non-small cell lung cancer
OS: overall survival
F3: coagulation factor III
